# Investigation on the Properties of Alkali-Activated Industrial Solid Waste and Excavated-Soil-Based Controlled Low-Strength Materials

**DOI:** 10.3390/ma18112474

**Published:** 2025-05-25

**Authors:** Chen Xu, Xiaolei Wang, Libo Liu, Yancang Li

**Affiliations:** College of Civil Engineering, Hebei University of Engineering, Handan 056038, China; c32120822@163.com (C.X.); liulibo@hebeu.edu.cn (L.L.); liyancang@hebeu.edu.cn (Y.L.)

**Keywords:** controlled low-strength material, excavated soil, industrial solid waste, fluidity, unconfined compressive strength

## Abstract

This study aims to address the challenge of backfill compaction in the confined spaces of municipal utility tunnel trenches and to develop an environmentally friendly, zero-cement-based backfill material. The research focuses on the excavation slag soil from a utility tunnel project in Handan. An alkali-activated industrial-solid-waste-excavated slag-soil-based controllable low-strength material (CLSM) was developed, using NaOH as the activator, a slag–fly ash composite system as the binder, and steel slag-excavated slag as the fine aggregate. The effects of the water-to-solid ratio (0.40–0.45) and the binder-to-sand ratio (0.20–0.40) on CLSM fluidity were studied to determine optimal values for these parameters. Additionally, the influence of excavated soil content (45–65%), slag content (30–70%), and NaOH content (1–5%) on fluidity (flowability and bleeding rate) and mechanical properties (3-day, 7-day, and 28-day unconfined compressive strength (UCS)) was investigated. The results showed that when the water-to-solid ratio is 0.445 and the binder-to-sand ratio is 0.30, the material meets both experimental and practical requirements. CLSM fluidity was mainly influenced by the excavated soil and slag contents, while NaOH content had minimal effect. The unconfined compressive strength at different curing ages was negatively correlated with the excavated soil content, while it was positively correlated with slag and NaOH content. Based on these findings, the preparation of “zero-cement” CLSM using industrial solid waste and excavation slag is feasible. For trench backfill projects, a mix of 50–60% excavated soil, 40–60% slag, and 3–5% NaOH is recommended for optimal engineering performance. CLSM is a new type of green backfill material that uses excavated soil and industrial solid waste to prepare alkali-activated materials. It can effectively increase the amount of excavated soil and alleviate energy consumption. This is conducive to the reuse of resources, environmental protection, and sustainable development.

## 1. Introduction

Global climate governance has prompted China to incorporate “carbon peaking and carbon neutrality” goals into its broader ecological civilization framework. Consequently, the development of a green, low-carbon, and circular economy has become a central national strategy. Relevant policies indicate that by 2025, all new urban and rural buildings must fully meet green building standards, raising demands for urban infrastructure development. The construction and industrial sectors, which account for over 50% of carbon emissions, urgently require a low-carbon transformation through technological innovation. As a key element of new urbanization, the urban underground comprehensive pipeline corridor plays a vital role in advancing green construction technologies. With the rapid advancement of infrastructure construction in China, most construction sites transport excavated soil to designated disposal sites for temporary storage [1,2,3]. Some sites directly use untreated excavated soil for backfilling; however, its strength often fails to meet engineering quality requirements. Therefore, addressing the challenges of storage, processing, and utilization of excavated soil and improving its efficient reuse have become key research focuses. In foundation pit and pipeline backfilling projects, construction is often constrained by narrow working spaces or inaccessible backfill areas. These limitations hinder the effectiveness of small tamping equipment, while manual compaction is inefficient and often fails to achieve the desired results [4,5,6].

Due to its excellent flowability and mechanical properties, controlled low-strength material (CLSM) has become a promising solution to these challenges. Under its self-weight, CLSM can autonomously fill voids and form a self-compacting structure, making it particularly suitable for construction in confined spaces. In recent years, extensive research has been conducted on CLSM material composition. Sheen, Y.N. et al. [7] found that substituting cement with slag effectively improved the fluidity of CLSM, significantly delayed the setting time, and notably reduced the compressive strength and pulse velocity at a fixed water–cement ratio. Mneina, A. et al. [8] found that incorporating treated oil sands waste (TOSW) enhanced the flowability of all CLSM, thereby reducing the water required to achieve the desired flowability. This also increased the compressive strength of CLSM containing both TOSW and fly ash. However, substituting fly ash with TOSW slightly decreased the strength of the CLSM. Zhang, J.X. et al. [9] found that the addition of 20–50% shotcrete accelerators reduced the percolation rate of CLSM but had no significant effect on its flowability. The flowability of CLSM could be improved by increasing the water-to-solids (W/S) or binder-to-recycled-fine-aggregate (B/R) ratios, which, respectively, increase or decrease the percolation rate. Do, T.M. et al. [10] found that by controlling the proportion of Fa-RmLG, a cementless binder composed of fly ash, lime, gypsum, and red mud, the engineering properties and environmental impacts of CLSM made with pond ash and Fa-RmLG met the required standards. This demonstrated the feasibility of producing CLSM using the developed Fa-RmLG binder. Qian, J.S. et al. [11] found that replacing part of the sand with excess excavated soil from a construction site in Shanghai reduced the strength and flowability of CLSM. Additionally, the consistency, water stability, and frost resistance of CLSM decreased as the soil content increased. While excessive soil can impair performance, excess excavated soil can be effectively utilized in CLSM with proper mix design. Tang, C.W. et al. [12] found the water-to-binder ratio to be the most significant factor influencing the fluidity of CLSM. The strength of CLSM containing water purification sludge (WPS) was lower than that of mixtures without WPS, especially when the WPS substitution for fine aggregate exceeded 20%, resulting in a significant reduction in strength. Ghanad, D.A. et al. [13] found that adding spruce residue reduced the compressive strength of CLSM, though the decrease was within acceptable limits. Good performance was maintained in CLSM with high cement content when spruce residue was included. The fine spruce residue also exhibited a filling effect, reducing porosity. Kim, Y.S. et al. [14] found that CLSM made with steelmaking slag (both raw and milled) exhibited good flowability and percolation, along with significantly improved UCS and water resistance. However, when the steelmaking slag content exceeded a certain level, thermal conductivity, UCS, and water resistance tended to decrease. Chen, H.J. et al. [15] found that when developing CLSM with stone sludge and lightweight aggregates, increasing the proportion of stone sludge roughly doubled the initial setting time. Additionally, the material cost per cubic meter of CLSM was lower than that of regular CLSM, reducing the cost by approximately 40%. Chen, T.X. et al. [16] found that when five different coal industry by-products (bottom ash (BA), fly ash, desulfurization gypsum, gasifier slag, and coal gangue) were used in mixtures with cement to prepare CLSM, optimizing the BA-to-fly-ash mass ratio and extending the ball-milling time of BA significantly reduced the fluidity and water permeability of the CLSM. Additionally, the 3-day UCS of the BA-containing CLSM was significantly higher than that of the BA-free group, while the 28-day UCS showed little change. Khadka, S.D. et al. [17] found that reducing the cement content and replacing it with an equal amount of fly ash (activated by an alkaline solution of NaOH and Na_2_SiO_3_) significantly improved the fluidity of the CLSM and reduced the setting time by more than 70%, compared to the conventional CLSM. Li, Y.C. et al. [18] found that increasing the iron ore tailings (IOT) content reduces the water required to achieve the desired fluidity of CLSM, significantly enhancing its unconfined compressive strength, cohesion, and friction angle. Mahamaya, M. et al. [19] found that in the preparation of CLSM using alkali-activated ferrochrome slag (FS), ground blast furnace slag, and fly ash, high molar ratios of potassium hydroxide (KOH) accelerated the alkali activation process, promoting the formation of alkaline aluminum/calcium silicate hydrates that bind the FS and produce CLSM with adequate strength. Qian, Y.F. et al. [20] found that controlling the length and amount of fibers significantly affected the 7-day UCS, 28-day UCS, and 28-day splitting strength of CLSM. Microscopic analysis further confirmed that the fibers reduced CLSM porosity by filling internal pores and interacting with hydration products, thereby forming a reticulated structure. Wan, X. et al. [21] found that the use of polycarboxylate superplasticizers (PCE) significantly improved the flowability of CLSM prepared from excavated waste soil. However, PCE negatively affected the early strength of CLSM, with this effect becoming positive as the curing time increased and hydration progressed. Wang, W.C. et al. [22] found that replacing natural fine aggregates with incineration bottom ash (IBA) had the greatest impact on the engineering properties of CLSM. IBA significantly improved the workability of CLSM, but the presence of CaSO_4_ negatively affected its setting time and mechanical properties. Treatment of IBA at 750 °C prior to use significantly enhanced the mechanical properties and reduced the setting time of IBA-CLSM. Mahamaya, M. et al. [23] found that cementless CLSM made from coal mine overburden (black shale) blended with varying proportions of alkali-activated ground granulated blast furnace slag (GGBS) and fly ash exhibited good flowability, compressive strength, and durability. The flow exceeded 200 mm; the relative flow area ranged from 2.06 to 7.70; the 28-day UCS value ranged from 0.48 MPa to 2.1 MPa; and the durability index ranged from 84.44% to 87.39%. Xiao, R. et al. [24] found that increasing the RM-to-GP ratio significantly reduced the flowability, prolonged the setting time, and impaired the mechanical properties of CLSM prepared with a ternary binder of slag, glass powder (GP), and red mud (RM), and crushed glass as aggregate. Compared to conventional CLSM made with cement and fly ash, the proposed formulations typically reduce carbon emissions and costs, highlighting their environmental sustainability and cost effectiveness. Xu, J.M. et al. [25] found that the fluidity of the excavated soil–cementitious CLSM increased roughly linearly with increasing water content, while its compressive strength decreased linearly. As cement content increased, the fluidity decreased roughly linearly, while the compressive strength increased linearly.

Conventionally, CLSM is produced using cement as the primary binder, supplemented with industrial solid waste such as iron slag, waste tires, waste glass, carbide slag, and titanium gypsum as partial replacements to promote solid waste recycling. However, studies on fully solid-waste-based CLSM remain limited, particularly regarding the use of excavated soil and industrial solid waste as raw materials. To address this gap, this study proposes a “zero-cement” CLSM, utilizing excavated soil, steel slag, slag, and fly ash as the primary raw materials. By adjusting the proportions of excavated soil, slag, and NaOH, the workability and mechanical properties of CLSM are investigated to meet backfilling requirements. This research aims to provide technical support for achieving a win-win solution in terms of resource conservation, environmental sustainability, and economic benefits.

## 2. Test Overview

### 2.1. Test Materials

**Figure 1:**
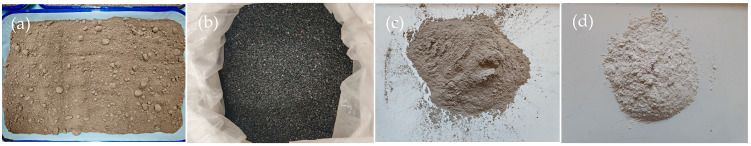
Raw materials: (**a**) Excavated soil; (**b**) Steel slag; (**c**) Slag; (**d**) Fly ash.

In this study, excavated soil and industrial solid waste (steel slag, slag, and fly ash) were used as raw materials to prepare controlled low-strength material (CLSM). The excavated soil was sourced from the underground comprehensive utility tunnel project in Handan, Hebei Province, and appeared yellowish-brown in color. After sampling, impurities were removed, and the soil was sieved through a 10 mm mesh to obtain the test material. Steel slag, slag, and fly ash were obtained from a factory in Hebei Province. The slag powder used is of S95 grade. A polycarboxylate superplasticizer was used as the admixture; NaOH served as the activator; and the water included distilled water and tap water. Figure 1 illustrates the raw materials.

**Figure 2:**
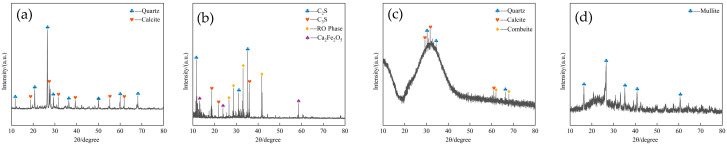
XRD analysis pattern of raw materials: (**a**) Excavated soil; (**b**) Steel slag; (**c**) Slag; (**d**) Fly ash.

**Figure 3:**
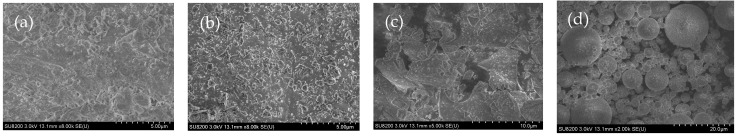
SEM micrographs of raw materials: (**a**) Excavated soil; (**b**) Steel slag; (**c**) Slag; (**d**) Fly ash.

The microstructure, mineralogical composition, and chemical composition of the materials were analyzed using X-ray fluorescence (XRF), X-ray diffraction (XRD), and scanning electron microscopy (SEM). Table 1 presents the primary chemical components, while Figure 2 depicts the mineralogical composition of the raw materials. Figure 3 illustrates the microstructure of the raw materials.

As shown in Table 1, the primary oxide in excavated soil is SiO_2_, followed by Al_2_O_3_ and CaO. The presence of Al_2_O_3_ contributes to the material’s pozzolanic activity. In steel slag, CaO is the dominant oxide, with SiO_2_ and Fe_2_O_3_ present in smaller amounts. Fly ash is mainly composed of SiO_2_, with Al_2_O_3_ as a secondary component. The combined content of SiO_2_ and Al_2_O_3_ exceeds 80% of the total composition, indicating that the strength development of fly ash largely depends on its pozzolanic reaction over time. The slag primarily contains SiO_2_ and CaO, along with a smaller proportion of Al_2_O_3_. The combined SiO_2_ and CaO content exceeds 70%, suggesting strong hydration and pozzolanic reactivity, making it a suitable alternative to cement.

As shown in Figure 2, the mineral phases of the excavated soil are predominantly quartz and calcite, with a higher quartz content, which provides enhanced skeletal support to the slag, while calcite contributes to improving the overall stability of the material. The mineral phases in steel slag include tricalcium silicate (C_3_S), dicalcium silicate (C_2_S), and calcium ferrite (Ca_2_Fe_2_O_5_), all of which are clinker minerals with potential hydration activity. Additionally, a certain amount of RO phase was detected, which exhibits low hydration activity and is considered an inert mineral. The mineral phase of fly ash is primarily mullite, which exhibits low reactivity. The mineral phases of slag are dominated by quartz and calcite, with a small amount of calcium rhodochrosite sodalite.

As shown in Figure 3, the excavated soil exhibits an irregularly stacked block structure with relatively few pores. Steel slag contains numerous irregular glassy structures with sharp edges and corners. Slag appears as fragmented particles, with some areas displaying fine pores or surface roughness. Fly ash consists of uniformly sized spherical particles with smooth surfaces. Its porous surface structure contributes to lower density, enhanced adsorption capacity, and improved reactivity.

### 2.2. Test Methods

**Figure 4:**
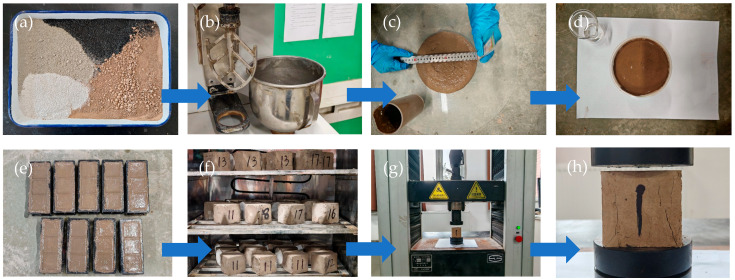
Test flowchart of CLSM: (**a**) Preparing materials; (**b**) Mixing materials; (**c**) Flowability test; (**d**) Bleeding rate test; (**e**) Specimens forming; (**f**) Curing specimens; (**g**) UCS test process; (**h**) After UCS test.

A review of the literature indicates that China has not yet established standardized test specifications for controlled low-strength material (CLSM). Therefore, this study draws on relevant international research and conducts tests with reference to applicable Chinese standards and regulations [26,27]. The flowability of CLSM slurry was tested following ASTM D6103-2017 (Standard Test Method for Flow Consistency of Controlled Low Strength Material) [28]. The bleeding rate test of CLSM slurry and the unconfined compressive strength (UCS) test of CLSM at different curing ages were conducted in accordance with the Highway Engineering Cement and Cement Concrete Test Regulations (JTG 3420-2020) [29]. The test flowchart of CLSM is displayed in Figure 4.

The tools required for the flowability test include a hollow cylindrical mold (75 mm in diameter and 150 mm in height), a glass plate (500 mm in diameter), a steel ruler, a spatula, a towel, and other necessary items. For the water secretion rate test, the required tools include a cushion block, a plastic graduated cylinder, an electronic balance, a rubber-tipped pipette, a beaker, a towel, and other related items. The equipment used for the unconfined compressive strength test is a microcomputer-controlled electronic universal testing machine. Table 2 presents the names of the test instruments, their model numbers, and the corresponding manufacturer information used in this study.

### 2.3. Determination of the Basic Water-to-Solid and Binder-to-Sand Ratios for CLSM

#### 2.3.1. Water-to-Solid Ratio

**Figure 5:**
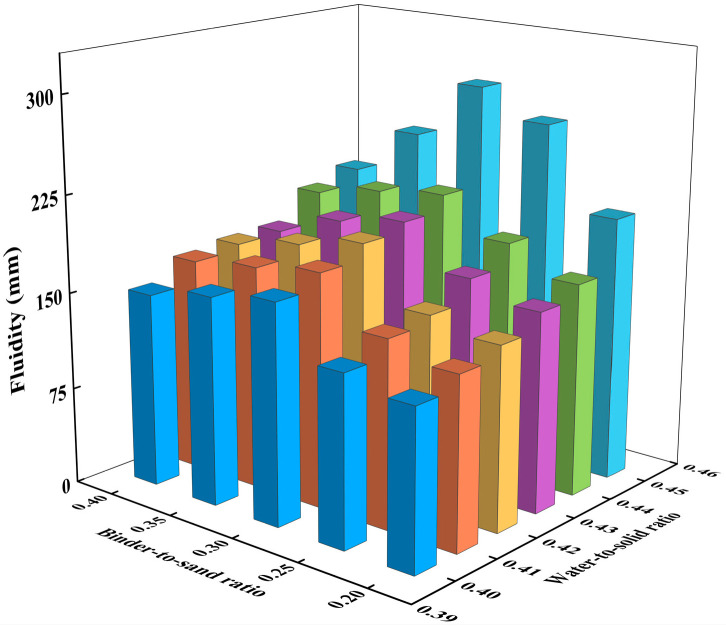
Experimental results on the effect of water-to-solid ratio and binder-to-sand ratio on CLSM flowability.

**Figure 6:**
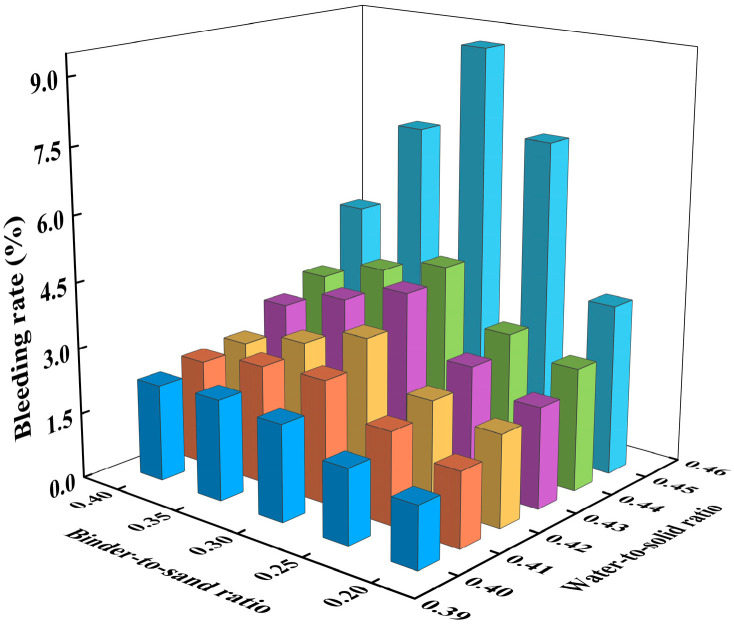
Experimental results on the effect of water-to-solid ratio and binder-to-sand ratio on CLSM bleeding rate.

Based on empirical data, the binder-to-sand ratio typically ranges from 0.2 to 0.4, while the water-to-solid ratio generally falls between 0.4 and 0.6. During the experiments, it was observed that when the water-to-solid ratio exceeded 0.45, bleeding occurred in the CLSM slurry. Therefore, in this section, the binder-to-sand ratio was set to 0.20, 0.25, 0.30, 0.35, and 0.40, and the fluidity of CLSM was investigated under water-to-solid ratios of 0.40, 0.41, 0.42, 0.43, 0.44, and 0.45. The mix design is shown in Table 3. Flowability and bleeding rate tests were conducted on CLSM with 30 different mix proportions, as shown in Table 3, and the corresponding results were obtained. These results are presented in Figure 5 and Figure 6.

Figure 5 illustrates the effect of the water-to-solid ratio and binder-to-sand ratio on flowability. As shown in Figure 5, the flowability of CLSM increases as the water-to-solid ratio increases under various binder-to-sand ratio conditions. When the water-to-solid ratio ranges from 0.40 to 0.44, the average flowability of CLSM under all binder-to-sand ratios is below 200 mm, which does not meet the design requirements for CLSM. The most significant change occurs when the water-to-solid ratio increases from 0.44 to 0.45. Specifically, at a water-to-solid ratio of 0.44, the average flowability of CLSM under different binder-to-sand ratios is 192.2 mm, classified as moderate flowability. However, at a water-to-solid ratio of 0.45, the average flowability increases to 242 mm, which is considered high flowability.

Figure 6 presents the effect of the water-to-solid ratio and binder-to-sand ratio on the bleeding rate. As shown in Figure 6, the bleeding rate of CLSM also increases as the water-to-solid ratio increases under different binder-to-sand ratio conditions. When the water-to-solid ratio is between 0.40 and 0.44, the average bleeding rates for the various binder-to-sand ratios are 1.96%, 2.34%, 2.66%, 3.18%, and 3.58%, all of which meet the specification requirement of a bleeding rate below 5%. However, when the water-to-solid ratio reaches 0.45, the average bleeding rate increases to 6.48%, which exceeds the maximum allowable limit of 5%.

In summary, based on the results of the flowability and bleeding rate tests, and considering the practical application requirements of CLSM, a water-to-solid ratio of 0.445 is determined to be optimal after comprehensive evaluation.

#### 2.3.2. Binder-to-Sand Ratio

**Figure 7:**
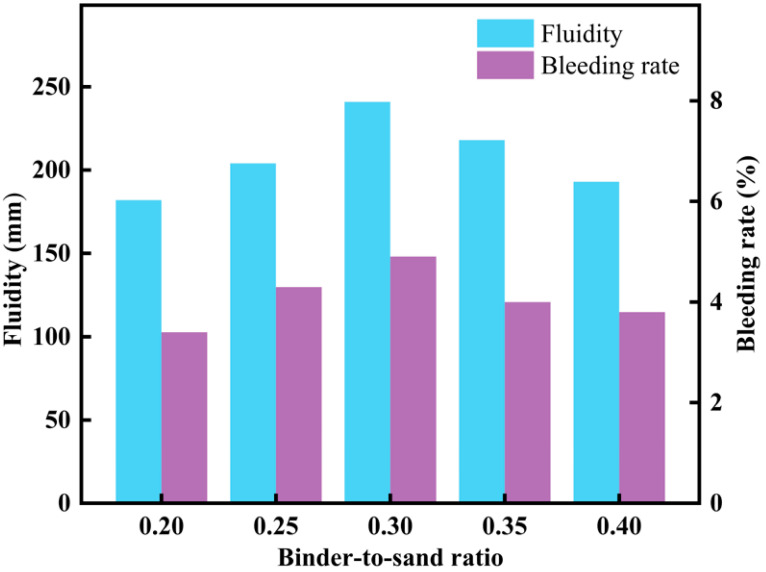
Experimental results on the effect of binder-to-sand ratio on CLSM flowability and bleeding rate.

The water-to-solid ratio was set to 0.445, and the fluidity and bleeding rate of CLSM were studied for binder-to-sand ratios of 0.20, 0.25, 0.30, 0.35, and 0.40. The mix design is shown in Table 4. Fluidity and bleeding rate tests were conducted on CLSM using the five different mix ratios shown in Table 4, and the results are presented in Figure 7.

Figure 7 illustrates the effect of binder-to-sand ratio on fluidity and bleeding rate. As shown in Figure 7, when the water-to-solid ratio is 0.445, the fluidity of CLSM increases initially and then decreases as the binder-to-sand ratio increases. The bleeding rate of CLSM follows a similar trend. This behavior can be explained by the fact that, as the binder-to-sand ratio increases, the proportion of fly ash and slag gradually increases, while the proportion of excavated soil and steel slag decreases. The increase in fly ash and slag helps fill the gaps between aggregates, forming a lubricating layer that improves the uniformity and fluidity of CLSM. However, when the binder-to-sand ratio exceeds 0.30 and continues to increase, the total amount of cementitious materials exceeds the optimal range for the CLSM material reaction system. At this point, excessive fly ash and slag cannot fully react, disrupting the balance of the reaction system. This leads to a decrease in viscosity, rather than the expected increase, significantly impairing fluidity. In line with the experimental results shown in Figure 4 and Figure 5, the fluidity and bleeding rate of CLSM also exhibit a similar pattern under different water-to-solid ratio conditions.

In summary, when the water-to-solid ratio is 0.445, the binder-to-sand ratios of 0.30 and 0.35 meet the requirements for CLSM. Given that the goal of this study is to maximize the utilization of excavated soil, reduce the preparation cost of CLSM, and meet green, environmentally friendly, and low-carbon development goals, the basic binder-to-sand ratio is set to 0.30.

### 2.4. Test Design

Based on the water-to-solid ratio of 0.445 and the binder-to-sand ratio of 0.30, as determined above, the effects of excavated soil content (45%, 50%, 55%, 60%, 65%), slag content (30%, 40%, 50%, 60%, 70%), and NaOH content (1%, 2%, 3%, 4%, 5%) on the working performance and mechanical properties of CLSM were studied. The performance was evaluated through fluidity tests, bleeding rate tests, and unconfined compressive strength tests. The fluidity and bleeding rate tests were repeated three times for each mix, and the results were averaged. The unconfined compressive strength tests were conducted at curing ages of 3, 7, and 28 days, with three specimens prepared for each age in each group. The compressive strength values were averaged, with the results reported to two decimal places (0.01 MPa). If the difference between the maximum or minimum value of the three specimens and the median value exceeded 15% of the median value, the median value was taken as the compressive strength of the group. If the difference between two test values and the median value exceeded 15%, the test results for that group were considered invalid, and new specimens were prepared. The mix designs are shown in Table 5, Table 6, and Table 7, respectively.

In this study, excavated soil content is defined as the mass ratio of excavated soil to fine aggregate; slag content is defined as the mass ratio of slag to cementitious material; and NaOH content is defined as the mass ratio of NaOH to cementitious material.

## 3. Results and Discussion

### 3.1. Excavated Soil Content Effect on CLSM Performance

#### 3.1.1. Effect of Excavated Soil Content on CLSM Fluidity

**Figure 8:**
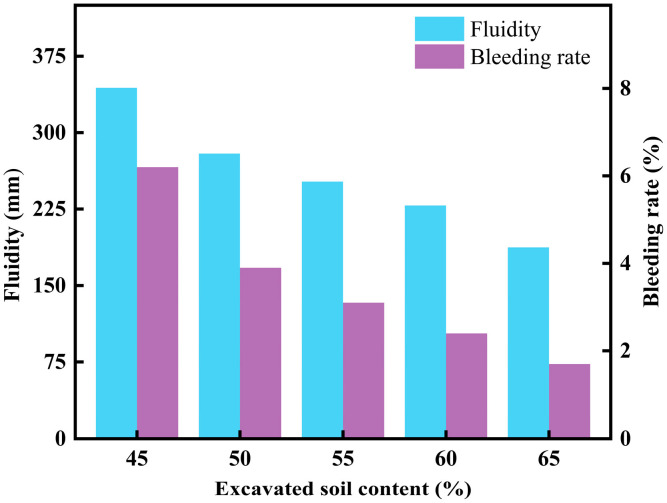
Experimental results on the effect of excavated soil content on CLSM flowability and bleeding rate.

Figure 8 illustrates the effect of excavated soil content on the fluidity and bleeding rate of CLSM. As the excavated soil content increases, the fluidity of CLSM gradually decreases. At an excavated soil content of 45%, the flowability reaches 344 mm. Within the range of 50–60% excavated soil content, the flowability decreases by approximately 25 mm for every 5% increase in content. When the excavated soil content reaches 65%, the flowability drops to 187.5 mm. The most significant reduction in flowability occurs when the content increases from 45% to 50%, decreasing sharply from 344 mm to 279.5 mm. At the same time, the bleeding rate of CLSM also decreases as the excavated soil content increases. When the excavated soil content is 45%, the bleeding rate is 6.2%. Within the range of 50–65% excavated soil content, the bleeding rate decreases by approximately 0.7% for every 5% increase in content. At 65% excavated soil content, the bleeding rate is reduced to 1.7%. The most significant reduction in bleeding rate occurs when the content increases from 45% to 50%, dropping from 6.2% to 3.9%.

This phenomenon can be attributed to the loose structure of excavated soil particles, which absorb more water during mixing. Unlike cement, which undergoes hydration upon water absorption to form a lubricating slurry, excavated soil does not immediately react with water. Instead, it leads to a “dry and stiff” CLSM system, resulting in decreased flowability as the excavated soil content increases. During the mixing process, it is evident that water absorption significantly increases after the addition of excavated soil, and the frictional resistance in the mixture also becomes more pronounced.

Overall, CLSM with a lower excavated soil content exhibits better fluidity compared to CLSM with a higher excavated soil content. When the excavated soil content is within the range of 50–60%, the fluidity of CLSM meets the specification requirements and satisfies practical engineering needs.

#### 3.1.2. Effect of Excavated Soil Content on CLSM Mechanical Properties

**Figure 9:**
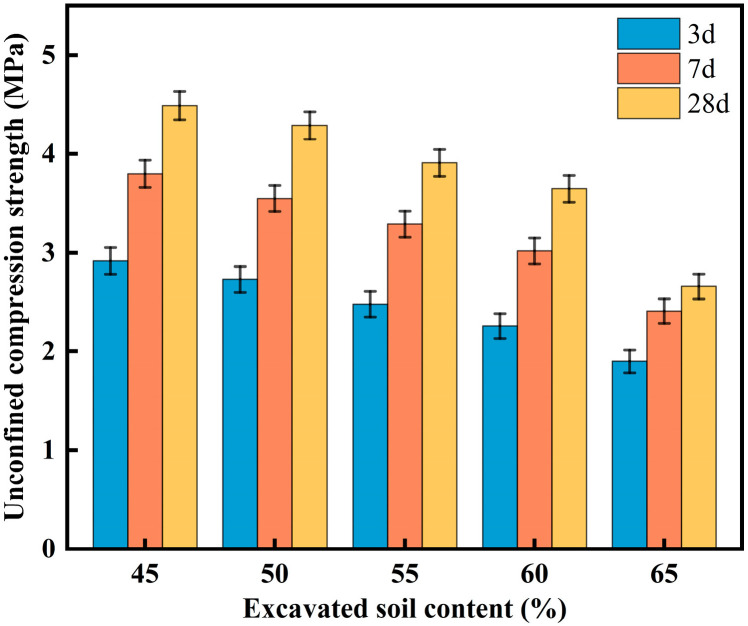
Experimental results on the effect of excavated soil content and curing ages on CLSM mechanical properties.

Figure 9 shows the effect of excavated soil content on the mechanical properties of CLSM. In Figure 9, it can be seen that by comparing the strength curves at different curing ages, the 7-day UCS increases by approximately 30% compared to the 3-day UCS, and the 28-day UCS increases by about 15% compared to the 7-day UCS. This indicates that the effect of early hydration on the unconfined compressive strength of CLSM is greater than that of later hydration, and the hydration rate of CLSM decreases and stabilizes in the later stages. As the proportion of excavated soil increases, the compressive strength of CLSM first decreases slowly and then decreases sharply. The reason for this can be explained by the fact that, as shown in Table 1, the CaO content in steel slag is 40.43%, while the CaO content in excavated soil is only 12.84%. As the content of excavated soil increases, the CaO content in CLSM decreases. Excessive excavated soil content leads to a shortage of active calcium compounds in the system, which affects the formation of effective polymer structures under alkaline conditions, thereby weakening the degree of hydration and reducing compressive strength. Xu, J.M. et al. found that the fluidity of the excavated soil–cementitious CLSM increased roughly linearly with increasing water content, while its compressive strength decreased linearly. As cement content increased, the fluidity decreased roughly linearly, while the compressive strength increased linearly [25]. Particularly without cement, when the excavated soil content is between 45% and 60%, the unconfined compressive strength of the test blocks at different curing ages is greater than 2.80 MPa but less than 8.40 MPa, which meets the relevant CLSM specifications. However, when the excavated soil content reaches 65%, the compressive strength of the samples at different curing ages significantly decreases compared to the 60% content, and the values fall below 2.80 MPa, which clearly does not meet the strength requirements for CLSM materials.

In summary, when the excavated soil content is between 50% and 60%, the working performance and mechanical properties of CLSM can meet both practical engineering requirements and the relevant specifications.

### 3.2. Slag Content Effect on CLSM Performance

#### 3.2.1. Effect of Slag Content on CLSM Fluidity

**Figure 10:**
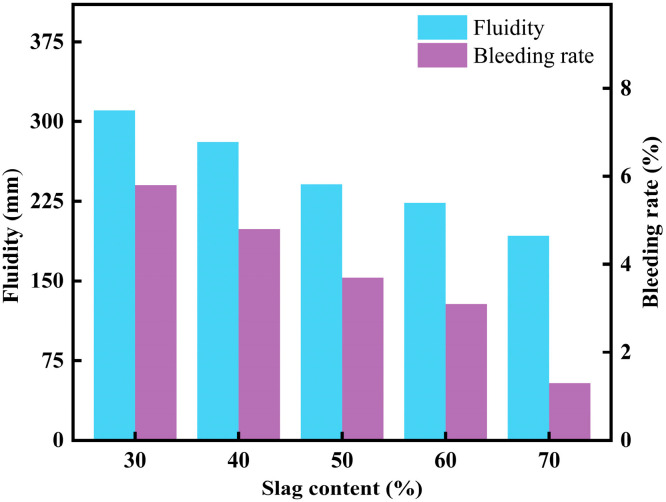
Experimental results on the effect of slag content on CLSM flowability and bleeding rate.

Figure 10 shows the effect of slag content on the working performance of CLSM. As seen in Figure 10, the fluidity and bleeding rate of CLSM are negatively correlated with slag content. When the slag content is 30%, the fluidity of CLSM reaches 310.5 mm, and the bleeding rate is 5.8%, both exceeding the upper application limits of 300 mm and 5%, respectively. When the slag content reaches 70%, the fluidity of CLSM drops to 192.5 mm, and the bleeding rate decreases to 1.3%, which does not meet the lower application limit of 200 mm. When the slag content is between 40% and 60%, the fluidity of CLSM ranges from 223.5 mm to 281.0 mm, and the bleeding rate ranges from 3.1% to 4.8%, both of which meet the specification requirements. In this range, both the fluidity and the bleeding rate meet the specification requirements. This behavior can be explained by the fact that when a large amount of fly ash is added to CLSM, it reacts with enough water, improving the material’s fluidity. This effect can be attributed to the ball-bearing effect of the fly ash particles. Sheen, Y. N et al. found that when developing slag-waste soil-based CLSM materials with slag as a cement replacement, an increase in slag content improves flowability and delays the setting time [7]. As shown in Figure 10, without cement, when the slag content is adjusted to 70%, there is a noticeable difference in fluidity characteristics compared to the 30% slag content. This is mainly because the low content of fly ash in the mix is insufficient to reduce the internal viscosity and particle friction within CLSM. As a result, the ball-bearing effect of the fly ash weakens, reducing the overall fluidity of the material.

In general, the working performance of CLSM with high slag content is lower than that with low slag content. CLSM with slag content ranging from 40% to 60% meets the specification requirements and satisfies practical engineering needs.

#### 3.2.2. Effect of Slag Content on CLSM Mechanical Properties

**Figure 11:**
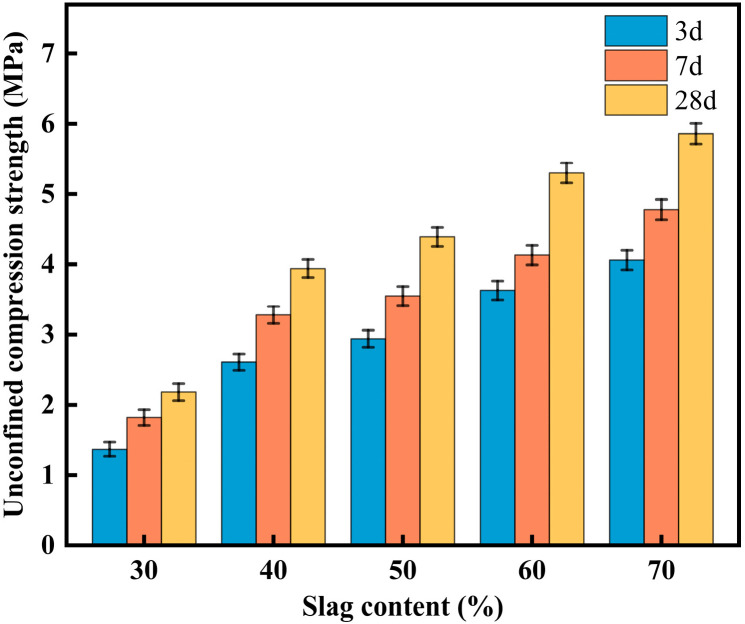
Experimental results on the effect of slag content and curing ages on CLSM mechanical properties.

Figure 11 shows the effect of slag content on the mechanical properties of CLSM. As observed in Figure 11, with the increase in slag content, the strength of CLSM increases rapidly at first and then rises more gradually.

When the slag content increased from 30% to 40%, the 3-day UCS of CLSM increased sharply from 1.37 MPa to 2.59 MPa. The 7-day UCS increased rapidly from 1.82 MPa to 3.46 MPa, and the 28-day UCS increased from 2.18 MPa to 3.94 MPa, with a growth rate exceeding 80%, indicating that the CLSM had a dense skeleton structure at this point. This can be explained by the fact that, as shown in Table 1, the CaO content in the slag is 35.46%, while the CaO content in fly ash is only 4.49%. As the slag content increases, the CaO content in the CLSM material increases correspondingly. The calcium compounds in CLSM not only generate hydrated calcium silicate gel phases under alkaline conditions, which act as the reaction core and accelerate hydration, but also promote the dissolution of fly ash and slag, thereby enhancing the pozzolanic reaction and improving material strength. When the slag content ranges from 40% to 70%, the unconfined compressive strength of the test blocks at different curing ages is greater than 2.80 MPa but less than 8.40 MPa, which meets the relevant CLSM specifications.

In summary, when the slag content is between 40% and 60%, the working performance and mechanical properties of CLSM meet both the practical engineering requirements and relevant specifications.

### 3.3. NaOH Content Effect on CLSM Performance

#### 3.3.1. Effect of NaOH Content on CLSM Fluidity

**Figure 12:**
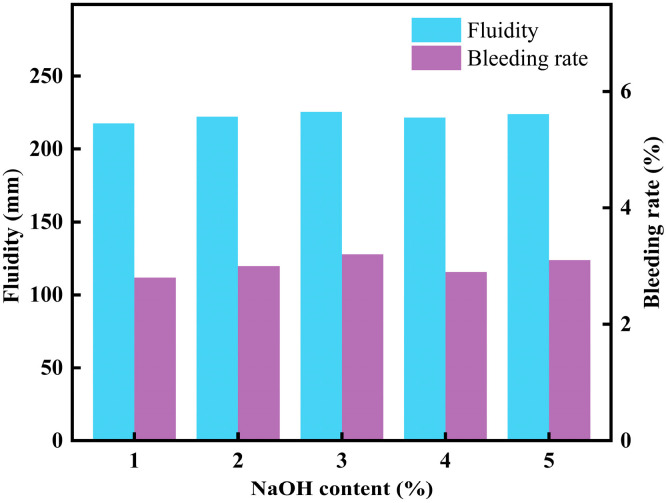
Experimental results on the effect of NaOH content on CLSM flowability and bleeding rate.

Figure 12 shows the effect of NaOH content on the working performance of CLSM. As shown in Figure 12, with the NaOH content varying from 1% to 5%, the fluidity of CLSM changes within the range of 208.5–290.5 mm, and the bleeding rate changes within the range of 3.3–4.7%. Khadka, S. D. et al. found that when fly ash activated by an alkaline solution formed with NaOH was used to replace an equal amount of cement to improve CLSM, the flowability of the modified CLSM was significantly enhanced compared to that of conventional cement-based CLSM [17]. Both the fluidity and bleeding rate meet the specification requirements. The fluidity change rate between adjacent NaOH content levels is approximately 1.7%, while the bleeding rate change rate is about 8.1%. These results indicate that NaOH content has no significant effect on the working performance of alkali-activated industrial solid waste–pipe gallery slag-soil-based CLSM.

#### 3.3.2. Effect of NaOH Content on CLSM Mechanical Properties

**Figure 13:**
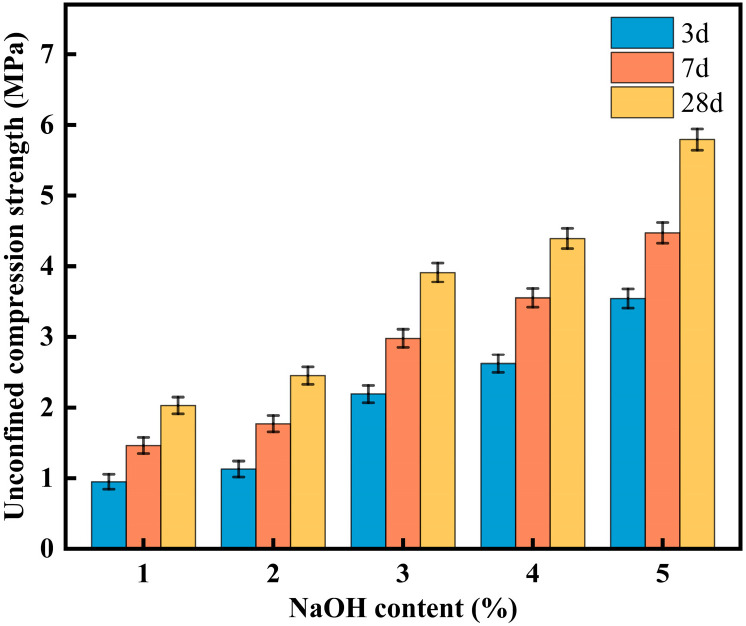
Experimental results on the effect of NaOH content and curing ages on CLSM mechanical properties.

Figure 13 shows the effect of NaOH content on the mechanical properties of CLSM. As seen in Figure 13, under the same curing age, the strength of CLSM increases with the increase in NaOH content. When the NaOH content is 1%, 2%, 3%, 4%, and 5%, the 28-day UCS of CLSM are 2.03 MPa, 2.45 MPa, 3.91 MPa, 4.39 MPa, and 5.79 MPa, respectively. As the NaOH content increases, the unconfined compressive strength increases by 21%, 93%, 116%, and 166%, indicating that the amount of NaOH significantly affects the development of CLSM’s unconfined compressive strength. Therefore, to design CLSM with varying strength to meet different mechanical conditions, controlling the NaOH content is crucial to satisfying different backfilling requirements. When the NaOH content is 5%, the 3-day UCS, 7-day UCS, and 28-day UCS of CLSM are 3.54 MPa, 4.47 MPa, and 5.79 MPa, respectively. With increasing curing age, the unconfined compressive strength increases by 26% and 64%, respectively, indicating that curing age also influences the development of CLSM’s unconfined compressive strength. The reason for this is that NaOH accelerates the hydration process within the material, which enhances the early strength. Notably, the strength increases rapidly when NaOH content rises from 2% to 3% and from 4% to 5%. In Figure 13, it can be observed that when the NaOH content is less than 3%, the compressive strength is below 2.80 MPa, which does not meet the design requirements.

In summary, when the NaOH content is between 3% and 5%, both the working performance and mechanical properties of CLSM meet practical engineering requirements and comply with the relevant specifications.

### 3.4. Microscopic Analysis

#### 3.4.1. Effect of Excavated Soil Content on CLSM Microscopic Characteristics

**Figure 14:**
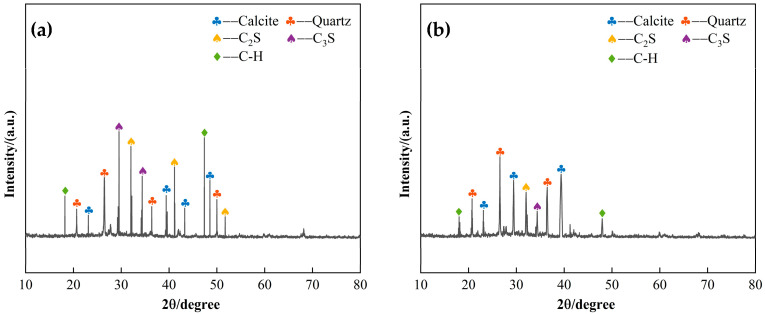
XRD patterns of the effect of excavated soil content on CLSM microscopic characteristics: (**a**) 45% excavated soil content; (**b**) 65% excavated soil content.

Two sets of CLSM specimens with 45% and 65% excavated soil content were subjected to X-ray diffraction (XRD) analysis after 28 days of curing, and the resulting XRD patterns are presented in Figure 14. Figure 14a illustrates the XRD pattern for the 45% excavated soil content (low content), while Figure 14b shows the XRD pattern for the 65% excavated soil content (high content). A comparison of the two patterns reveals that, in the low-content system, the C-H diffraction peaks are more pronounced, and the diffraction peaks for quartz and calcite are relatively weak. In contrast, the high-content system exhibits enhanced diffraction peaks for quartz and calcite, with distinct peaks for C_3_S and C_2_S, while C-H diffraction peaks are less pronounced. The analysis indicates that the hydration products primarily result from the hydration of C_3_S and C_2_S in steel slag in an aqueous or alkaline environment. C-H is formed by the directional dissolution of steel slag. The presence of quartz and calcite diffraction peaks in both systems suggests that these materials show limited reactivity in a non-highly alkaline environment, with only small amounts of SiO_2_ and Al_2_O_3_ potentially dissolving and contributing to the reactions. Consequently, quartz and calcite remain largely unreacted in their crystalline forms. In the low-content system, the C-H diffraction peaks are stronger than those of quartz and calcite, indicating that the synergistic hydration effect among steel slag, slag, fly ash, and NaOH is more pronounced at this content. When the excavated soil content increases to the high-content level, the system contains a larger proportion of quartz and calcite, which are less reactive. Additionally, the loose excavated soil particles reduce the available free water in the system, which hinders the hydration of steel slag. As a result, only a limited amount of C_3_S and C_2_S undergoes hydration. Figure 14b clearly shows that the diffraction peaks for C_3_S and C_2_S are more prominent in the high-content system, confirming that a high excavated soil content increases the proportion of low-reactivity inert materials within the system. This, in turn, adversely affects the structural integrity of the CLSM system. Moreover, the reduced free water content in the high-content system results in a lower formation of hydration products, which inhibits particle bonding and pore filling, ultimately leading to a decrease in the overall compressive strength of the CLSM.

**Figure 15:**
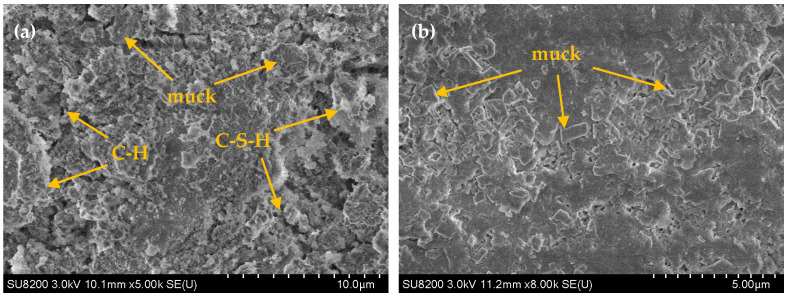
Corresponding microstructural images of the effect of excavated soil content on CLSM microscopic characteristics: (**a**) 45% excavated soil content; (**b**) 65% excavated soil content.

SEM analysis was performed on two sets of CLSM specimens with 45% and 65% excavated soil content after 28 days of curing, and the corresponding microstructural images are shown in Figure 15. Figure 15a illustrates the microstructure of the 45% excavated soil content (low content) specimens, while Figure 15b presents the microstructure of the 65% excavated soil content (high content) specimens. The SEM images reveal the inter-particle voids, hydration products (C-S-H and C-H), and unhydrated excavated soil particles. A comparison of the two images shows that, in the low-content system, there is a prominent presence of network-like C-S-H and flaky C-H, which aggregate into clusters on the surfaces of unhydrated excavated soil particles. These agglomerates, which vary in shape, facilitate particle inter-bonding, resulting in a more compact CLSM structure. In contrast, the high-content system exhibits a large number of irregular, flaky, or blocky particles with significant size variation and rough edges. Notably, the network-like C-S-H and flaky C-H hydration products are less evident. This can be attributed to the attachment of inert excavated soil particles to the surface of unhydrated steel slag particles. The accumulation of these inert particles on the surface of steel slag or excavated soil particles results in a denser spatial structure in the CLSM. However, the limited presence of hydration products in the high-content system prevents effective particle bonding, leading to reduced fluidity and compressive strength in the macroscopic tests. Moreover, as shown in Figure 15a, although some voids remain between particles in the low-content CLSM, these voids do not significantly compromise the overall strength. Instead, they enhance the fluidity of the CLSM slurry, which accounts for the superior fluidity observed in the low-content system compared to the high-content system.

In summary, the analysis of the phase composition and microstructure of CLSM, along with the macroscopic test results, confirms that using excavated soil alone as the fine aggregate in the CLSM system is not viable. It is essential to incorporate steel slag with higher reactivity as the fine aggregate to improve both fluidity and compressive strength.

#### 3.4.2. Effect of Slag Content on CLSM Microscopic Characteristics

**Figure 16:**
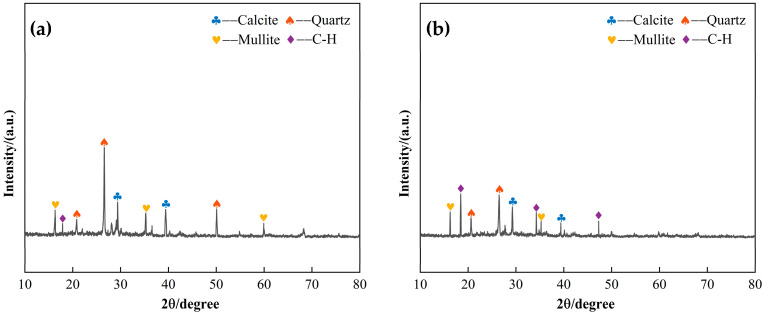
XRD patterns of the effect of slag content on CLSM microscopic characteristics: (**a**) 30% slag content; (**b**) 70% slag content.

Two sets of CLSM specimens with 30% and 70% slag content were subjected to XRD analysis after 28 days of curing, and the corresponding XRD patterns are shown in Figure 16. Figure 16a displays the XRD pattern for the 30% slag content (low content) CLSM, while Figure 16b shows the XRD pattern for the 70% slag content (high content) CLSM. A comparison of the two patterns reveals that, in the low-content system, the diffraction peaks for mullite, quartz, and calcite are more prominent. In contrast, in the high-content system, the C-H diffraction peaks are intensified, and the mullite diffraction peaks are significantly reduced. The analysis suggests that the observed hydration products result from the reaction between the amorphous Si and Al components, dissolved from slag and fly ash, with Ca^2+^ in the system, under the activation of NaOH. The presence of mullite diffraction peaks indicates that, due to the higher reactivity of slag compared to fly ash, the latter does not undergo complete reaction and remains as mullite in the system. In the low-content system, the fly ash content, being less reactive, is higher, while the slag content, which is more reactive, is lower. This leads to the formation of limited C-S-H and C-A-S-H hydration products under alkaline conditions, with the remaining mineral components maintaining their crystalline structure. As a result, the XRD patterns show pronounced diffraction peaks for mullite, quartz, and calcite. When the slag content increases, the diffraction peaks for quartz and calcite diminish, indicating that slag participates more actively in the reaction, generating amorphous C-S-H and C-A-S-H hydration products.

**Figure 17:**
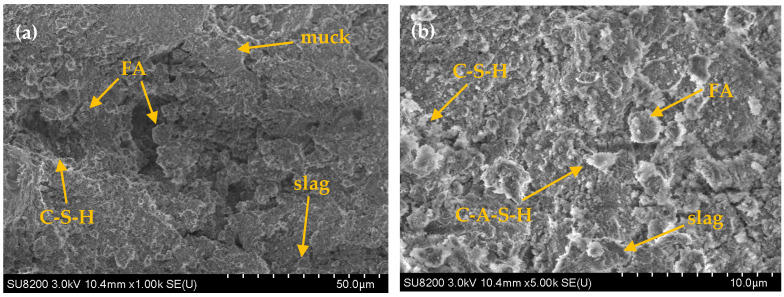
Corresponding microstructural images of the effect of slag content on CLSM microscopic characteristics: (**a**) 30% slag content; (**b**) 70% slag content.

SEM analysis was conducted on two sets of CLSM specimens with 30% and 70% slag content after 28 days of curing, and the corresponding microstructural images are presented in Figure 17. Figure 17a shows the microstructure of the 30% slag content (low dosage) specimens, while Figure 17b shows the microstructure of the 70% slag content (high dosage) specimens. The SEM images reveal inter-particle voids, hydrated C-S-H and C-A-S-H phases, as well as unhydrated raw material particles. A comparison of the two images reveals that, in the low-dosage system, the CLSM structure contains more voids, which can be attributed to the limited formation of reaction products, such as C-S-H and C-A-S-H. As a result, the bonding between particles is less pronounced, leading to a decrease in the overall structural integrity of the CLSM. While this structure results in lower compressive strength, the unhydrated fly ash particles, adhering to the surfaces of steel slag or slag particles, contribute to improved fluidity of the CLSM. In the high-dosage system, the voids in the CLSM structure are significantly reduced, and the particles are more tightly bonded. The increased amounts of slag and fly ash undergo more extensive reactions under alkaline conditions, resulting in the formation of flocculent hydration products, such as C-S-H and C-A-S-H, which bond the particles into agglomerates. As shown in Figure 17b, larger particles are observed on the surface of the agglomerates. These larger particles are likely unreacted slag particles that adhere to the agglomerates, reducing the void ratio and further enhancing the structural integrity of the CLSM.

In summary, based on the phase composition and microstructural analysis of CLSM, combined with macroscopic test results, it is viable to partially replace fly ash with slag as a cementitious material in the CLSM system. Moreover, the incorporation of an appropriate amount of slag enhances the compressive strength of the CLSM.

#### 3.4.3. Effect of NaOH Content on CLSM Microscopic Characteristics

**Figure 18:**
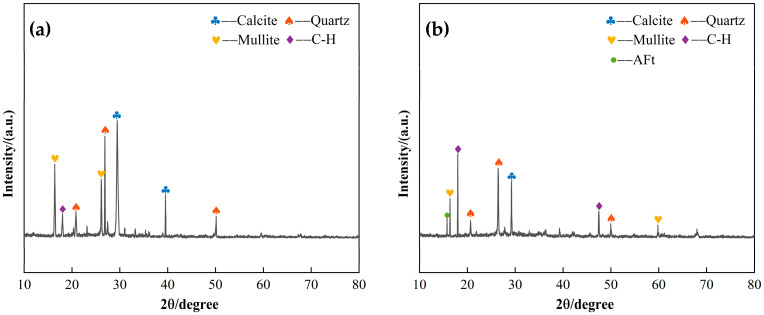
XRD patterns of the effect of NaOH content on CLSM microscopic characteristics: (**a**) 1% NaOH content; (**b**) 5% NaOH content.

Two sets of CLSM specimens with 1% and 5% NaOH content were subjected to XRD analysis after 28 days of curing, and the corresponding XRD patterns are shown in Figure 18. Figure 18a displays the XRD pattern for the 1% NaOH content (low content) system, while Figure 18b presents the XRD pattern for the 5% NaOH content (high content) system. A comparison of the two patterns reveals that, in the low-content system, the diffraction peaks of quartz, calcite, and mullite are more pronounced, indicating a higher content of these minerals. This is due to the substantial presence of quartz, calcite, and mullite in the raw materials, including excavated soil, slag, and fly ash. At 1% NaOH, the system is in a low-alkalinity environment, insufficient to fully activate the slag and fly ash. Consequently, only a portion of the slag reacts to form C-S-H and C-A-S-H gels, while the majority of fly ash and slag remain unreacted, with quartz, calcite, and mullite still present in the system. When the NaOH content is increased to 5%, the high-alkalinity environment fully activates the excavated soil, steel slag, fly ash, and slag. As a result, the diffraction peaks for quartz, calcite, and mullite weaken, while C-H diffraction peaks become more prominent. This suggests that in a high-alkaline environment, the slag releases amorphous Si and Al, and small amounts of SiO_2_ and Al_2_O_3_ from the slag dissolve and react with Ca^2+^ in the system to form C-S-H and C-A-S-H gels. Additionally, steel slag undergoes hydration reactions, producing C-H and C-S-H gels. These results indicate that increasing the NaOH content accelerates the hydration reactions between the raw materials in the CLSM system, leading to a greater variety and quantity of hydration products. This, in turn, improves the overall integrity of the CLSM and enhances its compressive strength.

**Figure 19:**
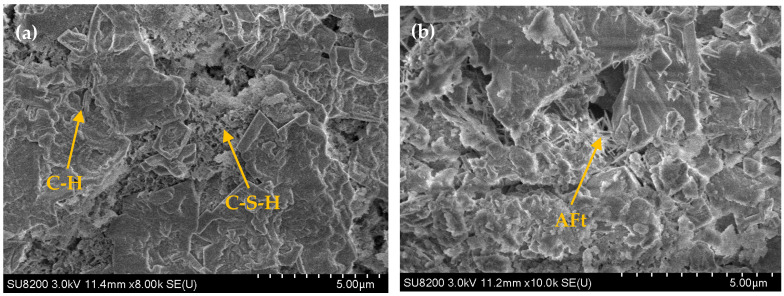
Corresponding microstructural images of the effect of NaOH content on CLSM microscopic characteristics: (**a**) 1% NaOH content; (**b**) 5% NaOH content.

SEM analysis was conducted on two sets of CLSM specimens with 1% and 5% NaOH content after 28 days of curing. The corresponding microstructural images are shown in Figure 19. Figure 19a presents the microstructure of the 1% NaOH (low content) specimens, while Figure 19b shows the microstructure of the 5% NaOH (high content) specimens. The SEM images reveal inter-particle voids, various hydration products, and unhydrated residue particles. In the low-content system, the microstructure exhibits abundant network-like C-S-H and flaky C-H, with C-H and C-S-H forming clusters on the surface of unhydrated residue particles. Although a small amount of gel binds the particles, significant voids remain, resulting in insufficient densification and lower compressive strength of the CLSM. In the high-content system, numerous irregular, flaky, or blocky particles with substantial size variation and rough edges are observed. These particles are primarily formed by hydration products such as C-H, C-S-H, and C-A-S-H attaching to the surface of unhydrated residue particles. The increased formation of C-S-H and C-A-S-H gels binds the fine aggregates, forming a denser and more stable structure, which enhances the integrity and compressive strength of the CLSM. Additionally, Figure 19b reveals the presence of a small number of needle-like structures between particles in the high-content CLSM. These structures are attributed to the rapid formation of C-A-H gels, which react with sulfate ions from the slag to form needle-like calcium aluminate (AFt). AFt exhibits swelling properties, and its growth fills the voids in the CLSM structure, further compacting the material and enhancing its strength. Although some voids remain in the high-content CLSM, they do not significantly affect the overall strength. Instead, they contribute to improved fluidity, which explains why the fluidity of the high-content CLSM is similar to that of the low-content CLSM.

In summary, based on the analysis of the phase composition and microstructure of CLSM, in conjunction with macroscopic test results, it is confirmed that adding NaOH and increasing its content within the CLSM system is effective. Additionally, to achieve higher compressive strength, slag, steel slag, and fly ash must be activated in a highly alkaline environment to enhance their reactivity.

## 4. Conclusions

In this paper, alkali-activated industrial solid waste excavated-soil-based CLSM was prepared using excavated soil, steel slag, slag, and fly ash as raw materials. The theoretical feasibility of preparing “no cement addition” CLSM with excavated soil and industrial solid waste was investigated. Through fluidity tests, bleeding rate tests, and unconfined compressive strength tests, the effects of water-to-solid ratio, binder-to-sand ratio, excavated soil content, slag content, and NaOH content on CLSM were studied in detail. Based on the test results, the following conclusions are drawn.

(1) Through the CLSM fluidity and bleeding rate tests, using a water-to-solid ratio of 0.445 and a binder-to-sand ratio of 0.30 as the basic design parameters for alkali-activated industrial solid waste excavated-soil-based CLSM, a CLSM with excellent working performance was prepared, exhibiting a fluidity greater than 200 mm and a bleeding rate of less than 5%.
(2) Based on the fluidity and bleeding rate tests, it was concluded that within the range of 45–65% excavated soil content, the fluidity of CLSM decreases as the excavated soil content increases. Similarly, within the range of 30–70% slag content, the fluidity decreases as the slag content increases. The addition of excavated soil significantly affects the fluidity of CLSM. Increasing the excavated soil content effectively reduces the bleeding rate. Excavated soil can be used as a feasible material to improve the stability and reduce the bleeding level of the proposed CLSM mixture.
(3) According to the unconfined compressive strength tests, it was found that within the range of 45–65% excavated soil content, the unconfined compressive strength of CLSM decreases with the increase in excavated soil content, while it increases with the increase in slag content within the range of 30–70%. The unconfined compressive strength also increases with the increase in NaOH content within the range of 1–5%. Furthermore, the unconfined compressive strength of CLSM increases as the curing age increases. The excavated soil content, slag content, and NaOH content are key factors influencing the unconfined compressive strength of CLSM.
(4) Through a comprehensive analysis of various factors affecting CLSM, it was concluded that a mix ratio of 50–60% excavated soil content, 40–60% slag content, and 3–5% NaOH content can be used to prepare a green and environmentally friendly CLSM that meets the practical engineering requirements. This study confirms the technical feasibility of using excavated soil to prepare CLSM and demonstrates a promising market application prospect.

This study focuses on the excavated soil generated by specific construction projects in the Handan area. It evaluates the feasibility of utilizing this soil for the preparation of controlled low-strength material (CLSM) and explores the variations in its key properties. Future research will investigate the workability, mechanical properties, and durability of CLSM prepared from excavated soil in different regions. The goal is to ensure the efficient recycling of excavated soil and to facilitate the widespread use of CLSM produced from such soil in various regions.

## Figures and Tables

**Table 1:** Chemical composition of raw materials (%).

Material	SiO_2_	Al_2_O_3_	CaO	Fe_2_O_3_	MgO	SO_3_	Others
Excavated soil	62.59	12.97	12.84	5.63	2.46	0.07	3.44
Steel slag	17.89	6.25	40.43	17.01	8.98	0.38	9.06
Slag	55.24	28.87	4.49	4.86	1.27	1.76	3.51
Fly ash	35.82	16.94	35.46	1.16	6.43	1.95	2.24

**Table 2:** Test instrument information.

Name	Model Number	Corresponding Manufacturer Information
Cement mortar mixing machine	JJ-20H	Tianjin Qingda Testing Instrument Manufacturing Co., Tianjin, China
Standard constant temperature and humidity curing box	YH-40B	Tianjin Luda Construction Instrument Co., Tianjin, China
Microcomputer-controlled electronic universal testing machine	WDW-100	Jinan Times Shijin Testing Instrument Co., Jinan, China
Electronic balance	PL3002	METTLER TOLEDO, Zurich, Switzerland

**Table 3:** Mix proportion and experimental design of water-to-solid ratio.

No.	Water-to-Solid Ratio	Binder-to-Sand Ratio	SS/g	ES/g	FA/g	Slag/g	Water/g
W40-C20	0.40	0.20	312.5	312.5	62.5	62.5	300.0
W40-C25	0.40	0.25	300.0	300.0	75.0	75.0	300.0
W40-C30	0.40	0.30	288.5	288.5	86.5	86.5	300.0
W40-C35	0.40	0.35	277.8	277.8	97.2	97.2	300.0
W40-C40	0.40	0.40	267.9	267.9	107.1	107.1	300.0
W41-C20	0.41	0.20	304.9	304.9	61.0	61.0	300.0
W41-C25	0.41	0.25	292.7	292.7	73.2	73.2	300.0
W41-C30	0.41	0.30	281.4	281.4	84.4	84.4	300.0
W41-C35	0.41	0.35	271.0	271.0	94.9	94.9	300.0
W41-C40	0.41	0.40	261.3	261.3	104.5	104.5	300.0
W42-C20	0.42	0.20	297.6	297.6	59.5	59.5	300.0
W42-C25	0.42	0.25	285.7	285.7	71.4	71.4	300.0
W42-C30	0.42	0.30	274.7	274.7	82.4	82.4	300.0
W42-C35	0.42	0.35	264.6	264.6	92.6	92.6	300.0
W42-C40	0.42	0.40	255.1	255.1	102.0	102.0	300.0
W43-C20	0.43	0.20	290.7	290.7	58.1	58.1	300.0
W43-C25	0.43	0.25	279.1	279.1	69.8	69.8	300.0
W43-C30	0.43	0.30	268.3	268.3	80.5	80.5	300.0
W43-C35	0.43	0.35	258.4	258.4	90.4	90.4	300.0
W43-C40	0.43	0.40	249.2	249.2	99.7	99.7	300.0
W44-C20	0.44	0.20	284.1	284.1	56.8	56.8	300.0
W44-C25	0.44	0.25	272.7	272.7	68.2	68.2	300.0
W44-C30	0.44	0.30	262.2	262.2	78.7	78.7	300.0
W44-C35	0.44	0.35	252.5	252.5	88.4	88.4	300.0
W44-C40	0.44	0.40	243.5	243.5	97.4	97.4	300.0
W45-C20	0.45	0.20	277.8	277.8	55.6	55.6	300.0
W45-C25	0.45	0.25	266.7	266.7	66.7	66.7	300.0
W45-C30	0.45	0.30	256.4	256.4	76.9	76.9	300.0
W45-C35	0.45	0.35	246.9	246.9	86.4	86.4	300.0
W45-C40	0.45	0.40	238.1	238.1	95.2	95.2	300.0

**Table 4:** Mix proportion and experimental design of binder-to-sand ratio.

No.	Water-to-Solid Ratio	Binder-to-Sand Ratio	SS/g	ES/g	FA/g	Slag/g	Water/g
C20	0.445	0.20	280.9	280.9	56.2	56.2	300.0
C25	0.445	0.25	269.7	269.7	67.4	67.4	300.0
C30	0.445	0.30	259.3	259.3	77.8	77.8	300.0
C35	0.445	0.35	249.7	249.7	87.4	87.4	300.0
C40	0.445	0.40	240.8	240.8	96.3	96.3	300.0

**Table 5:** Mix proportion and experimental design for different excavated soil content.

No.	Excavated Soil Content (%)	SS (g)	ES (g)	FA (g)	Slag (g)	NaOH (g)	Water (g)
E45	45	285.2	233.4	77.8	77.8	3.1	300.0
E50	50	259.3	259.3	77.8	77.8	3.1	300.0
E55	55	233.4	285.2	77.8	77.8	3.1	300.0
E60	60	207.4	311.1	77.8	77.8	3.1	300.0
E65	65	181.5	337.1	77.8	77.8	3.1	300.0

**Table 6:** Mix proportion and experimental design for different slag content.

No.	Slag Content (%)	SS (g)	ES (g)	FA (g)	Slag (g)	NaOH (g)	Water (g)
S30	30	259.3	259.3	108.9	46.7	3.1	300.0
S40	40	259.3	259.3	93.3	62.2	3.1	300.0
S50	50	259.3	259.3	77.8	77.8	3.1	300.0
S60	60	259.3	259.3	62.2	93.3	3.1	300.0
S70	70	259.3	259.3	46.7	108.9	3.1	300.0

**Table 7:** Mix proportion and experimental design for different NaOH content.

No.	NaOH Content (%)	SS (g)	ES (g)	FA (g)	Slag (g)	NaOH (g)	Water (g)
N1	1	259.3	259.3	77.8	77.8	1.6	300.0
N2	2	259.3	259.3	77.8	77.8	3.1	300.0
N3	3	259.3	259.3	77.8	77.8	4.7	300.0
N4	4	259.3	259.3	77.8	77.8	6.2	300.0
N5	5	259.3	259.3	77.8	77.8	7.8	300.0

## Data Availability

The original contributions presented in this study are included in the article. Further inquiries can be directed to the corresponding author.
